# Assessment of the factors influencing primary care physicians’ approach to vaccination of adult risk groups in Istanbul, Turkey

**DOI:** 10.7717/peerj.7516

**Published:** 2019-08-15

**Authors:** Fatma Yılmaz Karadağ, Zuhal Aydan Sağlam

**Affiliations:** 1 Department of Infectious Diseases and Clinical Microbiology, Istanbul Medeniyet University, Goztepe Training and Research Hospital, Istanbul, Turkey; 2 Department of Family Medicine, Istanbul Medeniyet University Goztepe Training and Research Hospital, Istanbul, Turkey

**Keywords:** Adult vaccination, Experience, Primary care physicians, Age, Physician own vaccination

## Abstract

**Background:**

We aimed to assess the factors influencing primary care physicians’ (PCPs) approach to adult vaccination in specific risk groups and evaluate the compliance to adult immunization guidelines.

**Methods:**

This cross-sectional study performed between January 2016 and April 2016 in İstanbul, Turkey. A questionnaire designed to obtain physicians’ demographical data, experience, immunization status, and attitude on prescribing or recommending vaccines for adults in the risk group. Healthy individuals older than 65 and patients suffer from chronic diseases or had splenectomy before are considered as a risk group. The questionnaire was sent via email to a randomly selected group of 1,500 PCPs. The data of 221 physicians who responded emails were recorded for statistical analysis.

**Results:**

Of the 221 participants (123 women, 98 men), the majority were aged 31–40 years. Their vaccination rates were 74.2% for hepatitis B, 54.3% for seasonal influenza, and 47.1% for tetanus. Among participants, the highest recommendation and prescription rate of adult vaccines was recorded in PCPs aged 31–40 years. In addition, PCPs with <10 years occupational experience were found to prescribe adult vaccines more frequently than PCPs with longer occupational experience.

**Conclusions:**

Primary care physicians with lower age and relatively less experience are more intent to prescribe adult vaccines to patients that are in risk groups. This result may be due to increased awareness of adult immunization among PCPs who had more recent medical training. However, many other factors could have caused this difference, including physicians’ approach to primary medical care.

## Introduction

Vaccination is a primary method for preventing infectious diseases. The Advisory Committee on Immunization Practices (ACIP) in the USA provides immunization recommendations for all age groups based on updated annual data (Kim, Riley & Hunter, 2018). Wide range of countries, including Turkey, refer to these recommendations to determine vaccination policies. Although childhood immunization programs are successfully implemented in the majority of the world (Murthy et al., 2017), adult immunization is still an important issue due to various reasons such as unwillingness to get vaccinated, concerns about the efficiency and side effects of vaccines, and inadequate knowledge about adult immunization and failure to recommend the necessary vaccines by the physicians (Lutz et al., 2018; Srivastav et al., 2018). In fact, adult vaccination has a vital role in the management of chronic diseases and immune deficiency; also it has a very important role in fighting against specific diseases which have increased the likelihood in some regions. Nevertheless, increasing the rate of adult immunization remains an important problem worldwide.

Primary care physicians (PCPs) play a critical role in childhood immunization and are also responsible for carrying out a targeted approach to immunization in adult patients (Campos-Outcalt et al., 2010; Gai & Gu, 2014; Leask, 2009). Therefore, PCPs’ attitude is vital to reach a desired rate of adult vaccination. According to the national vaccination schedule in Turkey, childhood vaccination rate in 2017 was 96%; however, data is limited in regard to national adult vaccination rates (Bora-Başara et al., 2018). Because there is no official data on the number of patients in risk groups, the number of healthy individuals aged 65 and over, and vaccination coverage rate among these groups; there is no clarified data on how to deal with the vaccination requirements of adult patients. Additionally, vaccination rates among risk groups are reportedly rather low in Turkey; however, these data have been obtained from various single-center studies (Table 1).

**Table 1:** Vaccination rates in risk groups in Turkey.

Risk groups	Influenza vaccination (%)	Pneumococcus vaccination (%)	References
COPD*	35.0	11.0	Uçar et al. (2015)
COPD*	40.0	10.0	Erer et al. (2013)
COPD*	37.9	13.3	Ozlu et al. (2019)
Splenectomy	–	75.0	Çalık et al. (2007)
≥65 years healthy people	26.8	3.1	Akman et al. (2014)
≥65 years healthy people	8.2	0.2	İlhan & Bakkaloğlu (2019)

**Note:**

* Chronic obstructive pulmonary diseases.

In Turkey, the major problem in adult immunization is the lack of a national health policy which is caused by the assumption that its economic burden would become a drain on the national budget (EKMUD Adult Immunization Working Group, 2009). Until recently, based on economic and financial reasons, adult immunization was overlooked in Turkey, except in certain special conditions and individual demands.

With this background in mind, we aimed to evaluate the factors influencing PCPs’ attitude to adult vaccination in the specified risk group. The risk group consists of three main categories: the patients older than 65, the patients suffer from the chronic obstructive pulmonary disease (COPD) or chronic liver disease, and the patients of those had splenectomy before.

We aimed to answer four main questions in our study: (1) What is the vaccination rate of physicians themselves? (2) What are the PCP’s rates of vaccination recommendation/prescription to adult patients in risk groups? (3) Is there any difference between PCPs that are vaccinated and those that are not, in terms of recommending vaccines to their patients? (4) What are the factors that are associated with the prescription of vaccines among PCPs? Since we aimed to evaluate the attitude of the physicians, both recommendations and prescriptions were considered as a positive attitude concerning adult vaccination.

## Materials and Methods

### Study design

This was a cross-sectional descriptive study that was conducted from January 20, 2016, to April 20, 2016, among PCPs who volunteered to participate in an online survey in Istanbul, Turkey. The survey was pretested for clarity and consistency among 18 PCPs and they were asked to comment on the clarity and language of the questions; after which appropriate corrections were performed. The questionnaire was anonymous and did not request any personal identifiers or information. The total number of PCPs in İstanbul was around 4,000. A comprehensive list of all PCPs in Istanbul was obtained from the Ministry of Health database. The questionnaire was sent via email. The survey requests were sent to a randomly selected group of 1,500 PCPs. Randomization was performed by using an online random selection program (random.org) without any specific rule set.

### Adult vaccine guidelines

The questionnaire was based on the adult immunization schedule recommendations that were published in 2015 by the ACIP of the Centers for Disease Control and Prevention (CDC) and national immunization guideline of the Turkish Ministry of Health.

According to the guideline, amongst the vaccines listed, tetanus-diphtheria (Td), influenza, pneumococcal, hepatitis vaccines are recommended to all risk groups. In addition, meningococcal vaccination is recommended for those who have undergone splenectomy.

Influenza is a disease which significantly increases health expenses due to its high burden of disease. It is actually a simple infection in which a simple vaccination can prevent consequences such as hospitalization and loss of labor. There is a risk of death, especially in risk groups. According to the World Health Organization, it is primarily recommended for pregnant women, health workers, children aged 6–59 months, older people over 65, and high-risk groups. In our country, it is recommended as an annual vaccine in all elderly patients and risk groups.

Although tetanus is an infectious disease, it is not transmitted from human to human and it is prevented by vaccination. Tetanus vaccine in our country is applied in the form of three doses in childhood which is suggested to be followed by vaccinations every 10 years thereafter.

Pneumococcal disease is also an important cause of morbidity and mortality that can be prevented by vaccination. Pneumococcal vaccination in adults is a cost-effective practice in the fight against pneumococcal disease. In Turkey, pneumococcal vaccination is recommended for all patients over the age of 65 and those with risk factors between 19 and 64 years of age (Şenol et al., 2018).

Hepatitis B infection is another vaccine-prevented disease, which is usually experienced during the childhood and may cause serious problems such as severe cirrhosis and liver failure. In our country, HBV vaccination is applied to risk groups and healthcare workers. In adults, application of HBV vaccine is performed by the standard 3-dose protocol (0, 1, and 6 months) in all adults, regardless of age.

Meningococcal infection is a disease with a high risk of mortality and the disease can be transmitted by human to human contact. The MEN ACWY-TT four-valent conjugated meningococcal vaccine (single dose 12 months) is used in Europe (including Turkey) for the immunization of adults and older children.

### Questionnaire design

The questionnaire was comprised of three sections. The first section included sociodemographic characteristics: age, gender, work area, professional experience, and the daily number of patients seen. The second section included questions about the personal immunization status of the physicians pertaining to hepatitis B, Td, and influenza vaccines. The third section evaluated when and how PCPs recommended and/or prescribed Td, pneumococcal, meningococcal, hepatitis A, hepatitis B, *Haemophilus influenza*, and influenza vaccines to healthy individuals aged ≥65 years and also patients with certain underlying diseases or conditions that put them into risk groups identified for vaccination, including diabetes mellitus (DM), COPD, chronic renal failure, chronic liver disease, history of splenectomy, and other vaccine-specific indications for each vaccine.

Since most adult vaccinations are not covered by the social security entity in Turkey, physicians usually tend to just recommend rather than prescribe adult vaccines. In some scenarios, patients reject to get vaccinated despite the physicians’ recommendations. Therefore, to distinguish prescriptions and recommendations was not always functional in evaluating the attitude of the physicians concerning adult vaccinations. When we were evaluating the relationship between PCPs’ vaccine history and attitude to adult vaccination, we prioritized the PCPs’ opinion and preferred “recommendation” criteria which are more inclusive and better represent the approach of the physician. However, when we were evaluating other factors (such as gender, age, experience etc.), we prioritized the “prescription” criteria which are more certain and represent the decision making of the physician and the patient as a whole.

### Statistical analysis

Data were analyzed using SPSS IBM 22.0 (SPSS Inc., Chicago, IL, USA) statistics software. The distribution of data was assessed using the Kolmogorov–Smirnov test. In the results, data were presented as frequency and percent values for count data and as mean ± SD (standard deviation) for normally distributed variables. Correlation analyses were performed to identify any possible correlation between participants’ demographic features, experience, vaccination history, and vaccine recommendation/prescription rates. Correlations tests between the questioned factors and rate of positive attitude to adult vaccination were performed using the Pearson Chi-square and Fisher’s exact tests. The level of statistical significance was set at *p* < 0.05.

### Ethical

Ethics committee approval for the study was obtained from the Istanbul Medeniyet University Göztepe Training and Research Hospital Ethics Committee on January 26, 2016, with the approval number: 2016/0015.

## Results

A total of 221 PCPs that responded to the survey were included in the study. The response rate was very low (14.7%). According to gender, 123 of 221 subjects were female and 98 were male. In regard to age, 61.5% (*n* = 136) of the PCPs were younger than 40, and 99 were in the 31–40 age group. The number of PCPs with ≤10 years of professional experience was 72% (*n* = 161). Sociodemographic characteristics of the participants are shown in Table 1; 89% of the participants were working at city centers and 95% (*n* = 210) had over 3,000 registered patients. The mean number of daily visits was 35 ± 12.2 for patients aged >18 years; 15 ± 8.9 for patients aged >65 years; and 15 ± 10.4 for the risk groups. Among the participants, 72.4% (*n* = 160/221) were following the regulations of the Ministry of Health to keep up to date with current information on adult immunization. The percentage of PCPs that recommended tetanus, influenza, and HBV vaccinations to patients aged ≥65 years (in accordance with Ministry of Health recommendations) were 65.6%, 71.2%, and 62.5%, respectively.

Since the response rate was far lower than expected, a sufficient amount of data concerning some factors and some vaccine types could not be gathered. Nevertheless, the main goals of the study were achieved. Findings concerning our four main questions are given below:

### What is the vaccination rate of physicians themselves?

Among the 221 participants in our study, the rates of tetanus, influenza, and HBV vaccinations were 47.1%, 54.3%, and 74.2%, respectively. When PCPs were compared, females were found to have a higher percentage for influenza vaccination (*p* = 0.035), while males and females were similar for tetanus and HBV vaccination. In terms of age groups, the 31–40 years age group had higher rates of tetanus, influenza, and HBV vaccination compared to other age groups.

### What are the PCP’s rates of vaccination recommendation/prescription to adult patients in risk groups?

In terms of recommendations; 73.8%, 76.5%, and 68.8% of PCPs reported that they recommended tetanus, influenza, and HBV immunization respectively to those in risk groups. The percentage of PCPs that recommended the other vaccines in the questionnaire to patients identified to be in risk groups were as follows: 33.3% for hepatitis A, 69.7% for pneumococcal, 37.6% for meningococcal, and 33.1% for *Haemophilus influenza* vaccines.

### Is there any difference between PCPs that were vaccinated and those that were not, in terms of prescribing or recommending vaccines to their patients?

When we compared vaccine prescription rates with regard to PCPs’ own vaccination rates, we found that recommendation frequencies of vaccines to risk groups were higher among physicians who had chosen to vaccinate themselves.

Primary care physicians who had had HBV vaccination were found to recommend HBV and influenza vaccines to their patients more often, while the same relationship was not apparent in tetanus vaccine recommendation.

Primary care physicians who had had influenza vaccinations were found to recommend influenza and tetanus vaccines to their patients more often, while the same relationship was not seen in HBV vaccine recommendation.

Primary care physicians who had had tetanus vaccinations were found to recommend HBV and influenza vaccines to their patients more often, while the same relationship was not seen in tetanus vaccine recommendation (Table 2).

**Table 2:** Primary care physicians’ own vaccination status and vaccine prescription rates.

		Vaccine prescription								
		HBV			Influenza			Tetanus		
Physicians’ status		No (%)	Yes (%)	*p* value	No (%)	Yes	*p* value	No (%)	Yes (%)	*p* value
HBV	No	34 (59.6)	35 (21.3)	0.001	38 (37.6)	31 (25.8)	0.060	25 (21.4)	44 (42.3)	0.001
	Yes	23 (40.4)	129 (78.7)		63 (62.4)	89 (74.2)		92 (78.6)	60 (57.7)	
Influenza	No	28 (49.1)	24 (14.6)	0.001	36 (35.6)	16 (13.3)	0.001	12 (10.3)	40 (38.5)	0.001
	Yes	29 (50.9)	140 (85.4)		65 (64.4)	104 (86.7)		105 (89.7)	64 (61.5)	
Tetanus	No	10 (17.5)	48 (29.3)	0.083	19 (18.8)	39 (32.5)	0.021	31 (26.5)	27 (26.0)	0.928
	Yes	47 (82.5)	116 (70.7)		82 (81.2)	81 (67.5)		86 (73.5)	77 (74.0)	

### What are the factors that affect the prescription of vaccines among PCPs?

#### Gender

When recommendations of PCPs were evaluated, there were no differences in terms of gender for PCPs’ recommendation of tetanus (*p* = 0.359), influenza (*p* = 0.388), HBV (*p* = 0.137), HAV (*p* = 0.830), pneumococcal (*p* = 0.728), meningococcal (*p* = 0.259), and *H. influenza* (*p* = 0.560) vaccinations. Female PCPs were found to prescribe pneumococcal and tetanus vaccines at a significantly higher rate compared to males, while influenza prescription rates were similar.

#### Age and experience

There was no difference in PCPs recommendations for tetanus (*p* = 0.371), HAV (0.394), pneumococcal (*p* = 0.166), and *H. influenza* (*p* = 0.597) vaccinations in terms of age groups, while PCPs in the 31–40-year age group recommended the Influenza (*p* = 0.001), HBV (*p* = 0.003), and meningococcal (*p* = 0.029) vaccines more often than their counterparts in other age groups.

In terms of age groups, younger PCPs had higher vaccine prescription rates (pneumococcal, influenza, and tetanus) compared to those over the age of 40 years; however, interestingly, the 31–40 years age group had significantly higher prescription rate compared to both younger (24–30 years) and older (>40 years) PCPs. Experience as a PCP was also found to be effective on the prescription rates of the pneumococcal and tetanus vaccines. Interestingly, those with 0–10 years of experience were found to have higher pneumococcal and tetanus vaccine prescription rates compared to those with >10 years of experience as a PCP, while there was no difference for the influenza vaccine. When PCPs that were family medicine specialists and those that were general practitioners working as family physicians were compared, we found no differences in vaccine prescription rates.

#### Patient number

When PCPs were compared in regard to the number of patients assigned to them, the only difference was found in terms of tetanus vaccine prescription, showing that those with lower patient numbers had a higher tendency to prescribe the tetanus vaccine (Table 3).

**Table 3:** Demographic features of primary care physicians and vaccine prescription rates.

	Pneumococcus vaccine prescription			Influenza vaccine prescription			Tetanus vaccine prescription		
	No (%)	Yes (%)	*p* value	No (%)	Yes (%)	*p* value	No (%)	Yes (%)	*p* value
Gender									
Male	76 (49)	23 (34.8)	0.052	64 (44.4)	35 (45.5)	0.886	49 (44.5)	50 (45)	0.006
Female	79 (51)	43 (65.2)		80 (55.6)	42 (54.5)		61 (55.5)	61 (55)	
Age									
24–30 years	17 (11)	20 (30.3)	0.001	15 (10.4)	22 (28.6)	0.001	4 (3.6 )	33 (29.7)	0.001
31–40 years	64 (41.3)	35 (53.0)		59 (41)	40 (51.9)		43 (39.1)	56 (50.5)	
>40 years	74 (47.7)	11 (16.7)		70 (48.6)	15 (19.5)		63 (57.3)	22 (19.8)	
Experience									
0–10 years	107 (69)	55 (83.3)	0.028	100 (69.4)	62 (80.5)	0.076	66 (60)	96 (86.5)	0.001
>10 years	48 (31)	11 (16.7)		44 (30.6)	15 (19.5)		44 (40)	15 (13.5)	
Specialty									
Family physician	33 (21.3)	16 (24.2)	0.629	36 (25)	13 (16.9)	0.166	23 (20.9)	26 (23.4 )	0.653
General practitioner	122 (78.7)	50 (75.8)		108 (75)	64 (83.1)		87 (79.1)	85 (76.6 )	
The number of patients assigned to the physician									
>4,000	67 (43.2 )	20 (30.3)	0.072	59 (41)	28 (36.4)	0.504	43 (39.1)	44 (39.6 )	0.957
3,000–4,000	88 (56.8% )	46 (69.7)		85 (59)	49 (63.6)		67 (60.9 )	67 (60.4)	

## Discussion

In this study, we found that younger PCPs are more intent on prescribing adult vaccines to patients in certain risk groups including those with COPD, chronic liver disease, and prior splenectomy. We also found that PCPs with lower experience were prescribing adult vaccines at a slightly higher proportion than their counterparts with higher experience.

Adult immunization is strongly recommended in patients with several diseases which alter immunity, including DM, COPD, and chronic liver diseases. According to the Global Initiative for Chronic Obstructive Lung Disease 2011 COPD guidelines, both influenza and pneumococcal vaccines are suggested for all patients with COPD in order to lower exacerbation rates and associated mortality (Vestbo et al., 2013). In addition, international guidelines recommend HAV and HBV vaccinations for disease prevention in patients with chronic liver diseases (Fiore, Wasley & Bell, 2006). Vaccination for influenza, pneumococcus, and hepatitis B is also recommended in patients with DM. In patients with prior splenectomy, vaccination against S. pneumonia, *N. meningitidis*, *H. influenza* type b, and influenza virus are strongly recommended as these patients are vulnerable to invasive infections caused by encapsulated bacteria (Bonanni et al., 2017).

Healthcare professionals are also considered to be at high risk of vaccine-preventable diseases, particularly those caused by blood- and air-borne pathogens. The vaccines recommended for healthcare professionals include influenza, measles-mumps-rubella, hepatitis B, varicella, Td, meningococcal, and hepatitis A (Kim, Riley & Hunter, 2018). In the current study, the percentage of participants who had had vaccinations for HBV, tetanus, and influenza were comparable to global averages reported in various studies (Castilla et al., 2013; Opstelten et al., 2008; Pulcini et al., 2013; Semaille et al., 2006). However, there are countries in which physician vaccination rates have reached desired levels (Cowan et al., 2006; Paya et al., 2013) which shows that there is still much that can be done to increase the immunization awareness of PCPs. In our study, female PCPs were found to have significantly higher rates of influenza vaccination and PCPs aged 31–40 years had higher immunization rates for all three vaccines. In terms of recommending vaccines to patients, gender was found to have no effect, while PCPs aged 31–40 were found to be more likely to recommend influenza, HBV, and meningococcal vaccines to patients in respective risk groups.

In the current study, PCPs with lower age (<40) and lesser experience (<10 years) were more likely to prescribe adult vaccines in certain risk groups. However, in a previous study, Asma et al. (2016) reported that vaccination compliance was higher in PCPs with increased age and higher experience. Another study investigating healthcare professionals’ attitude to vaccination revealed that participants with higher experience were more likely to get vaccinated (Ali et al., 2018). Although our findings are conflicting with the results of many previous studies, some possible explanations could be derived regarding this controversy.

First of all, there is limited data investigating the role of age and occupational experience on vaccine prescription rates. As mentioned above, up-to-date knowledge is of great importance to understand the preventative role of vaccines in the adult population. However, physicians with higher age and experience almost always have higher patient counts and are probably unable to follow current scientific data on immunization due to time limitations, while younger PCPs with relatively less experience are more up to date, which may be due to their more recent medical school training. In our study, most of the participants stated that they were following the Health Ministry’s regulations as the sole resource of information on vaccination, which may indicate the absence of effort or facilities for reaching more current data on adult vaccination. In addition, lack of motivation in terms of keeping up to date with medical changes may also be a factor negatively impacting the knowledge of PCPs, as there is currently no “board examination” aimed at regularly evaluating the primary healthcare knowledge of PCPs. Such an examination—with a clear and concise approach—may motivate physicians to follow recent developments and guidelines other than those proposed by the Ministry of Health. Under these circumstances—in which PCPs do not, or cannot keep up to date with current guidelines—information gained at medical school and Ministry of Health guidelines become the only source for their future occupational practice, which constitutes an answer for the question why younger and relatively less-experienced PCPs more frequently prescribe adult vaccines. However, many other factors may contribute to the difference observed in this study. Our results by no means prove these assumptions. Future studies would benefit from evaluating other parameters associated with primary care in order to come to an accurate conclusion on this matter.

We also evaluated whether there was a relationship between personal vaccination and prescription of vaccines to patients in risk groups. Although adult vaccination is crucial especially in certain risk groups, the importance of adult vaccination might be underestimated by healthcare professionals, particularly PCPs who are always in communication with their registered populations and primarily responsible for vaccination (Albano et al., 2014; Yassi et al., 1994). A systematic review written by Wilson et al. (2015) reported that, lower recommendation by healthcare workers, reservations about vaccine safety, belief that vaccines are not needed or effective, and low knowledge about vaccines and their cost were the most prominent factors influencing low vaccination rates. A survey study conducted in Australia on adult patients admitted to the emergency department reported that 84.6% of those who received vaccinations trusted their family physicians and considered the vaccines that they recommended (Sandhofer et al., 2017). Although conflicting results exist, a nationwide survey conducted in Germany, which showed that although a high proportion of the healthcare workers were influenza vaccinated, many of them had not recommended or prescribed influenza vaccine to their patients (Wortberg et al., 2009). Furthermore, several studies have shown that PCPs’ vaccine recommendations to patients are significantly associated with their own immunization attitudes (Godoy et al., 2015; Nichol & Zimmerman, 2001; Poland, 2010; Verger et al., 2012). In a recent study carried out in Italy, investigators have shown a clear decline in the frequency of health care workers’ vaccination status with increasing age—with lowest rates in health care workers aged between 51 and 60 years (Gilardi et al., 2018). Therefore, we believe that the knowledge of a PCP and their subsequent approach to patients are the major factors that influence the vaccination of adults. In the light of our findings and data obtained from the literature, we believe that increasing the awareness of PCPs in terms of vaccine effectiveness and indications may contribute significantly to the immunization of adults in risk groups.

### Limitations

Our study has some limitations that must be stated. First, the response rate to the questionnaire was quite low. This may have been due to the possibility that non-responders may be less concerned about adult immunization; therefore, causing a bias towards the inclusion of PCPs that already recognized the importance of the topic. Second, we only used the prescription rate of vaccines to evaluate the attitude and intention of PCPs regarding adult immunization; however, prescription rates may have also been associated with various other PCP- and patient-related factors. Third, the number of participants may be considered relatively low, possibly impacting statistical analysis. Finally, the PCPs included in the study may not be in the risk groups of the majority of vaccines evaluated; however, we believe we addressed this problem by only evaluating the rates of HBV, influenza, and tetanus vaccinations among PCPs (which are highly recommended for physicians). However, we believe our study in its current form adds important data to the literature, especially regarding the associations between vaccination behaviors of PCPs and factors such as physician age and occupational experience.

## Conclusion

Our study revealed that younger PCPs (especially those aged between 31 and 40 years) and those with relatively less experience were more likely to prescribe adult vaccines, especially in certain risk groups that were predisposed to vaccine-preventable diseases. Our results indirectly emphasized the role of physicians’ knowledge level on immunization. According to the results of the present study, we suggest that educational programs should be carried out for PCPs aged over 40 years and those with more than 10 years of occupational experience, in order to improve their knowledge and understanding of adult immunization.

## Supplemental Information

10.7717/peerj.7516/supp-1 Supplemental Information 1 Adult vaccination.

10.7717/peerj.7516/supp-2 Supplemental Information 2 Adult vaccination encoding.

10.7717/peerj.7516/supp-3 Supplemental Information 3 Questionnaire for adult immunization.

## Additional Information and Declarations

### Data Availability

The following information was supplied regarding data availability: Raw measurements are available in the Supplemental Files.
