# Supplementary material for: Assessment of the factors influencing primary care physicians’ approach to vaccination of adult risk groups in Istanbul, Turkey
Source: PeerJ. 2019 Aug 15;7:e7516. doi: 10.7717/peerj.7516 (PMC6698375; doi:10.7717/peerj.7516)
Supplement: Supplemental Information 2 [file peerj-07-7516-s002.docx]

**DATA ENCODİNG**

**1-Gender :** Female: 0, male: 1

**2-Age:**

20-25 yr: 1, 26-30 yr: 2, 31-40 yr: 3, 41-50 yr: 4, 51-60 yr: 5, > 60 yr : 6

**3-Experience**

- 1. yr: 1, 1-5 yr: 2, 6-10 yr : 3, 11-15 yr: 4, 16-20 yr: 5, > 20 : 6

**4-Speciality :** Family physician : 1, Practitioner : 2

**5- Numbers of patients following up**

< 2000: 1, 2001-3000: 2, 3001-4000: 3, > 4000: 4

**6- Following current information on adult immunization**

IF following regulation of the Ministry Health : 1, if the others: 0

**7- Have you been vaccinated ?**

No: 1, Yes : 2

**8- Have you vaccinated Hepatitis B ?**

No: 1, Yes: 2, Resolved hepatitis B: 3, Chronic hepatitis B: 4

**9- Do you recommend vaccination ?**

No: 0, Yes: 1

**10- Have you prescribed vaccines last month ?**

No: 0, Yes: 1

Chronic obstrutive pulmonary disease: COPD

Chronic renal failure : CRL

Chronic liver disease: CLD
