# Supplementary material for: Assessment of the factors influencing primary care physicians’ approach to vaccination of adult risk groups in Istanbul, Turkey
Source: PeerJ. 2019 Aug 15;7:e7516. doi: 10.7717/peerj.7516 (PMC6698375; doi:10.7717/peerj.7516)
Supplement: Supplemental Information 3 [file peerj-07-7516-s003.pdf]

Evaluation of the knowledge and attitude of primary care physicians towards adult immunization

Dear Participant,

I am going to give you information and invite you to be part of this research. This questionnaire was organized by the collaboration of two departments (Department of Microbiology and Infectious Diseases; Department of Family Medicine) at Istanbul Medeniyet University Göztepe Training and Research Hospital. With this questionnaire we aim to investigate the levels of knowledge, attitude and behavior of primary care physicians about adult immunization. The results of this research, which you will contribute on a voluntary basis as a primary care physician, will be evaluated in accordance with scientific ethics. Data collected will be evaluated individually and will be kept confidential. Completing the questionnaire is based on volunteerism and this form will be sent to the e-mail address of the people.

We thank you for accepting to participate in the study and hope that you will answer all the questions in the survey sincerely. We thank you for your contributions and the time you have given us.

First Section

1. Gender

- ○ Female
- ○ Gender

2. Age (years)

○ 20-25 ○ 26-30 ○ 31-40 ○ 41-50 ○ 51-60 ○ > 60

3. How long have you been working ? (years)

○ 0-1 ○ 1-5 ○ 6-10 ○ 11-15 ○ 16-20 ○ >20

4. You are working as a………..?

○ Family physician ○ Practitioner

5. In which area do you work?

- ○ 1.Region : Major cities (provinces with metropolitan municipality)
- ○ 2.Region : Other cities
- ○ 3.Region : District
- ○ 4.Region: Town
- ○ 5. Region: Village

6. How many patients do you have to follow up?

○ < 2000 ○ 2001-3000 ○ 3001-4000 ○ >4000

7. How many patients do you visit daily at the age of 18 and over? [ ]

8. How many patients do you visit daily at the age of 65 and over? [ ]

9.How many patients do you visit with diabetes mellitus, chronic obstructive pulmonary disease, chronic renal failure, chronic liver disease or history of splenectomy daily ?

[ ]

Second Section

10. Have you been vaccinated for tetanus ?

○ Yes ○ No

11. Have you been vaccinated for Hepatitis B ?

○ Yes ○ No ○ Resolved hepatitis B ○ Chronic hepatitis B

12. Have you been vaccinated for influenza this year ?

○ Yes ○ No

13. How do you follow current information on adult immunization?

○ Regulations of the Ministry of Health
○ Advisory Committee on Immunization Practices (ACIP) Recommendations
○ Journal about Vaccination
○ Vaccination companies visits / promotion
○ Others

Third Section

14. Which vaccines do you recommend for healthy adults aged 65 and over?

○ Tetanus	○ Yes	○ No
○ Influenza	○ Yes	○ No
○ Hepatitis B	○ Yes	○ No
○ Hepatitis A	○ Yes	○ No
○ Pneumococcal	○ Yes	○ No
○ Meningococcal	○ Yes	○ No
○ H.influenza	○ Yes	○ No

15. Which vaccines do you recommend for patients with diabetes mellitus ?

○ Tetanus	○ Yes	○ No
○ Influenza	○ Yes	○ No
○ Hepatitis B	○ Yes	○ No
○ Hepatitis A	○ Yes	○ No
○ Pneumococcal	○ Yes	○ No
○ Meningococcal	○ Yes	○ No
○ H.influenza	○ Yes	○ No

16. Which vaccines do you recommend for patients with chronic obstructive pulmonary disease?

○ Tetanus	○ Yes	○ No
○ Influenza	○ Yes	○ No
○ Hepatitis B	○ Yes	○ No
○ Hepatitis A	○ Yes	○ No
○ Pneumococcal	○ Yes	○ No
○ Meningococcal	○ Yes	○ No
○ H.influenza	○ Yes	○ No

17. Which vaccines do you recommend for patients with chronic renal failure ?

○ Tetanus	○ Yes	○ No
○ Influenza	○ Yes	○ No
○ Hepatitis B	○ Yes	○ No
○ Hepatitis A	○ Yes	○ No
○ Pneumococcal	○ Yes	○ No
○ Meningococcal	○ Yes	○ No
○ H.influenza	○ Yes	○ No

18. Which vaccines do you recommend for patients with chronic liver disease?

○ Tetanus	○ Yes	○ No
○ Influenza	○ Yes	○ No
○ Hepatitis B	○ Yes	○ No
○ Hepatitis A	○ Yes	○ No
○ Pneumococcal	○ Yes	○ No
○ Meningococcal	○ Yes	○ No
○ H.influenza	○ Yes	○ No

19.Which vaccines do you recommend for patients with history of splenectomy ?

○ Tetanus	○ Yes	○ No
○ Influenza	○ Yes	○ No
○ Hepatitis B	○ Yes	○ No
○ Hepatitis A	○ Yes	○ No
○ Pneumococcal	○ Yes	○ No
○ Meningococcal	○ Yes	○ No
○ H.influenza	○ Yes	○ No

20. For which group of patients have you prescribed tetanus vaccine last month?

○ Healthy adults age ≥ 65 years	○ Yes	○ No
○ Chronic renal failure	○ Yes	○ No
○ Chronic liver disease	○ Yes	○ No
○ Chronic obstructive pulmonary disease	○ Yes	○ No
○ Diabetes mellitus	○ Yes	○ No
○ Splenectomy	○ Yes	○ No

21. For which group of patients have you prescribed influenza vaccine last month?

○ Healthy adults age ≥ 65 years	○ Yes	○ No
○ Chronic renal failure	○ Yes	○ No
○ Chronic liver disease	○ Yes	○ No
○ Chronic obstructive pulmonary disease	○ Yes	○ No
○ Diabetes mellitus	○ Yes	○ No
○ Splenectomy	○ Yes	○ No

22. For which group of patients have you prescribed hepatitis B vaccine last month?

○ Healthy adults age ≥ 65 years	○ Yes	○ No
○ Chronic renal failure	○ Yes	○ No
○ Chronic liver disease	○ Yes	○ No
○ Chronic obstructive pulmonary disease	○ Yes	○ No
○ Diabetes mellitus	○ Yes	○ No
○ Splenectomy	○ Yes	○ No

23. For which group of patients have you prescribed hepatitis A vaccine last month ?

Healthy adults age ≥ 65 years	Yes	No
Chronic renal failure	Yes	No
Chronic liver disease	Yes	No
Chronic obstructive pulmonary disease	Yes	No
Diabetes mellitus	Yes	No
Splenectomy	Yes	No

24. For which group of patients have you prescribed pneumococcal vaccine last month?

Healthy adults age ≥ 65 years	Yes	No
Chronic renal failure	Yes	No
Chronic liver disease	Yes	No
Chronic obstructive pulmonary disease	Yes	No
Diabetes mellitus	Yes	No
Splenectomy	Yes	No

25. For which group of patients have you prescribed meningococcal vaccine last month?

Healthy adults age ≥ 65 years	Yes	No
Chronic renal failure	Yes	No
Chronic liver disease	Yes	No
Chronic obstructive pulmonary disease	Yes	No
Diabetes mellitus	Yes	No
Splenectomy	Yes	No

26. For which group of patients have you prescribed *H. Influenza* tybe b vaccine last month?

Healthy adults age ≥ 65 years	Yes	No
Chronic renal failure	Yes	No
Chronic liver disease	Yes	No
Chronic obstructive pulmonary disease	Yes	No
Diabetes mellitus	Yes	No
Splenectomy	Yes	No
